# Esophageal Squamous Papilloma and Papillomatosis: Current Evidence of HPV Involvement and Malignant Potential

**DOI:** 10.3390/cancers17142404

**Published:** 2025-07-20

**Authors:** Miriana Mercurio, Roberto de Sire, Paola Campagnoli, Marco Dal Fante, Linda Fazzini, Luciano Guerra, Massimo Primignani, Maria Giuseppina Tatarella, Mauro Sollai, Sandro Ardizzone, Roberta Maselli

**Affiliations:** 1 Endoscopy Unit, Gastroenterology Department, IRCCS Humanitas Research Hospital, Via Alessandro Manzoni 56, 20089 Rozzano, Italy; miriana.mercurio@humanitas.it (M.M.); roberto.desire@humanitas.it (R.d.S.); roberta.maselli@hunimed.eu (R.M.); 2 Department of Biomedical Sciences, Humanitas University, Via Rita Levi Montalcini 4, Pieve Emanuele, 20072 Milan, Italy; 3 Gastroenterology and Digestive Endoscopy Unit, Humanitas San Pio X, Via Francesco Nava 31, 20159 Milan, Italy; paola.campagnoli@sanpiox.humanitas.it (P.C.); marco.dal_fante@sanpiox.humanitas.it (M.D.F.); linda.fazzini@sanpiox.humanitas.it (L.F.); luciano.guerra@sanpiox.humanitas.it (L.G.); massimo.primignani@sanpiox.humanitas.it (M.P.); maria.tatarella@sanpiox.humanitas.it (M.G.T.); 4 Pathology Unit, IRCCS Humanitas Research Hospital, Via Alessandro Manzoni 56, 20089 Rozzano, Italy; mauro.sollai@humanitas.it

**Keywords:** Esophageal squamous papilloma, esophageal papillomatosis, human papillomavirus (HPV), endoscopy, malignant potential, surveillance

## Abstract

Human papillomavirus (HPV) is a recognized oncogenic agent in several epithelial malignancies, though its role in esophageal squamous lesions remains unclear. Esophageal squamous papilloma and papillomatosis are rare, often benign lesions, but increasing evidence suggests possible associations with high-risk HPV genotypes and a non-negligible risk of dysplasia and malignant transformation. This narrative review summarizes current evidence on epidemiology, clinical features, histopathology, and diagnostic approaches, emphasizing advanced endoscopic imaging techniques that improve lesion detection and characterization. Management relies primarily on complete endoscopic resection with histological and virological evaluation. While small, non-dysplastic solitary lesions may not require routine surveillance, multifocal or high-risk HPV-positive cases warrant closer follow-up. Standardized HPV testing and long-term prospective studies are needed to better define the oncogenic potential and inform surveillance and treatment strategies.

## Simple Summary

Esophageal squamous papilloma (ESP) and papillomatosis are rare esophageal lesions traditionally considered benign, yet emerging evidence suggests a possible association with high-risk human papillomavirus (HPV) infection and a potential risk of malignant progression. In this review, we summarize current evidence on their pathogenesis, clinical features, diagnostic approaches, and management strategies, with particular focus on the role of HPV. We also discuss the contribution of advanced endoscopic imaging in detecting lesions with dysplastic changes. Given the limited and heterogeneous data available, further research is urgently needed to clarify the oncogenic potential of these lesions and to optimize surveillance and treatment protocols.

## 1. Introduction

Human papillomavirus (HPV) is a well-established etiologic agent in various epithelial malignancies, including cervical, anogenital, and oropharyngeal cancers [1]. In recent decades, increasing attention has been directed toward its potential involvement in esophageal squamous pathology. Among the esophageal mucosal alterations possibly linked to HPV infection, squamous esophageal papilloma (ESP) and papillomatosis represent rare but increasingly investigated entities. Although traditionally considered benign, emerging evidence suggests a potential association with high-risk HPV (hr-HPV) genotypes and a non-negligible risk of dysplasia or malignant transformation [2,3].

This review aims to synthesize current knowledge on HPV-related esophageal lesions, focusing on their epidemiology, clinical, and histopathological features, HPV association, recurrence rates, malignant potential, diagnostic approaches—including advanced endoscopic imaging—and current treatment and surveillance strategies.

## 2. Human Papillomavirus Infection

### 2.1. Biology and Oncogenesis 

Human papillomavirus is a small, non-enveloped, double-stranded DNA virus belonging to the Papillomaviridae family. It infects basal keratinocytes of the skin and mucosal surfaces and is one of the most prevalent sexually transmitted infections worldwide. The estimated lifetime risk of HPV infection is approximately 50% for both men and women [4]. Established risk factors include a high number of lifetime sexual partners, early onset of sexual activity, and co-infections with other sexually transmitted pathogens, including HIV [5,6]. In children, transmission may occur through hand-to-mouth contact with infected non-genital lesions, perinatally via vaginal delivery, or, less commonly, in utero via ascending maternal infection [7,8].

Although increasing evidence links HPV to the development of esophageal squamous papillomas and papillomatosis, the precise mechanisms of viral transmission to esophageal mucosa remain unclear. Morris [9] hypothesized that contamination of the upper aerodigestive tract could occur as neonates pass through an HPV-infected birth canal. This theory is supported by data from juvenile-onset recurrent respiratory papillomatosis (RRP), where HPV types 6 and 11 are commonly involved [10]. Although rare, concurrent involvement of the esophagus and the laryngotracheal tract has been reported, particularly in pediatric patients or those with perinatal HPV exposure [11,12], suggesting a potential susceptibility related to shared exposure of the aerodigestive mucosa.

Additional case reports have suggested that HPV transmission may occur through sexual contact [13], direct mucosal exposure [14], or perinatal infection [15].

Certain sexual behaviors have also been associated with increased risk of HPV transmission to the upper aerodigestive tract, potentially contributing to carcinogenesis. These include early onset of sexual activity, multiple sexual partners, unprotected oro-genital contact, and male-to-male intercourse. In a cohort of men who have sex with men, Mistry et al. [16] demonstrated a significant association between oral sex and oral/oropharyngeal HPV infection (*p* = 0.0038). Similarly, Dalla Torre et al. [17] found that both a high number of vaginal (*p* = 0.0001) and oral (*p* < 0.0001) sexual partners were significantly associated with oral HPV positivity in young adults aged 18 to 30 years.

Despite these data, there is currently no statistically significant evidence supporting a higher prevalence of HPV-related esophageal lesions among patients with concurrent cervical or penile HPV-associated diseases.

Most HPV infections are asymptomatic, and approximately 90% of individuals clear the virus spontaneously. In about 10% of cases, however, the infection persists, significantly increasing the risk of malignant transformation. In immunocompetent adults, most transient mucosal infections resolve within 8–14 months, while persistence beyond 24 months is exceedingly rare outside immunosuppressed or HIV-positive settings [18]. Over 100 HPV genotypes have been identified and are classified as high-risk or low-risk based on their oncogenic potential. Twelve high-risk genotypes (types 16, 18, 31, 33, 35, 39, 45, 51, 52, 56, 58, and 59) are classified as carcinogenic by the International Agency for Research on Cancer (IARC), while eight additional types (types 26, 53, 66, 67, 67, 70, 73, and 82) are considered “probably” or “possibly” carcinogenic. Among them, HPV types 16 and 18 are the most frequently associated with high-grade dysplasia and cancer [19,20].

Persistent infection with hr-HPV occurs through viral entry via microabrasions, allowing the virus to reach the basal layer of the epithelium. Initially, the viral genome remains episomal and replicates in basal cells. Malignant progression is often associated with viral DNA integration into the host genome, a key event that drives the overexpression of the viral oncoproteins E6 and E7 [21,22]. These oncoproteins inactivate critical tumor suppressors. E6 promotes the degradation of p53, and E7 inactivates the retinoblastoma protein (pRb). The inactivation of pRb leads to the upregulation of the cyclin-dependent kinase inhibitor p16INK4a (p16) [23,24], which is frequently used as a surrogate marker for oncogenic HPV activity [25].

### 2.2. HPV Detection Methods

Several methodologies are available for detecting HPV in tissue specimens and exfoliated cells. While these techniques are well-established in gynecologic and oropharyngeal oncology, their optimal application in esophageal pathology remains debated.

Commonly used methods include polymerase chain reaction (PCR), in situ hybridization (ISH), and immunohistochemistry (IHC) for p16 [26,27]. PCR-based assays offer high sensitivity and specificity for detecting HPV DNA [27]. Although ISH is less sensitive, it provides spatial context by localizing viral DNA within the tissue architecture, which enhances histopathological interpretation [26]. p16 overexpression detected by IHC is a widely accepted surrogate marker of transcriptionally active HPV in cervical and oropharyngeal cancer due to its link to E7-mediated pRb pathway disruption [25,28]. However, in esophageal squamous cell carcinoma (ESCC), p16 expression has not consistently correlated with the presence of HPV DNA [29,30]. Therefore, p16 cannot be considered a reliable surrogate marker of HPV-driven oncogenesis in ESCC. PCR and ISH remain the most frequently used detection methods for HPV in esophageal tissue [30,31]. 

Reported HPV detection rates in esophageal samples vary widely, from 10% to 80%, depending on the technique used, geographic differences, and patient selection. PCR-based methods generally yield higher sensitivity than ISH [30,31].

In the histopathologic assessment of HPV-related esophageal papillomas and papillomatosis, koilocytic changes—such as nuclear enlargement, irregular nuclear membranes, and perinuclear clearing—are occasionally observed [2,32]. However, their prevalence has not been systematically quantified, and these features are neither pathognomonic nor consistently present. Their diagnostic value increases when accompanied by molecular confirmation using PCR or ISH.

This variability in histologic and virologic findings highlights the need for standardized diagnostic criteria and detection protocols in future studies of HPV-associated esophageal lesions.

## 3. HPV-Related Esophageal Lesions

Among the various esophageal abnormalities, esophageal squamous papilloma and papillomatosis have emerged as the primary lesions potentially associated with HPV infection. Although relatively rare, these entities have attracted increasing attention due to their suspected viral etiology and potential for malignant transformation.

### 3.1. Esophageal Squamous Papilloma

Esophageal squamous papilloma is a rare, benign epithelial tumor first described by Adler et al. in 1959 [33]. Since then, the understanding of ESPs has evolved primarily through case reports and small series. 

The lesion is an uncommon finding during esophagogastroduodenoscopy (EGD), with a reported prevalence ranging from 0.01% to 0.45% [34,35,36,37]. Pediatric prevalence appears comparable to that in adults, approximately 0.08% [38]. Despite its rarity, ESP prevalence appears to be increasing: Pantham et al. reported a rise from 0.13% in 2000 to 0.57% in 2013 [39]. 

Due to the low number of studies, demographic data remain limited. Available evidence suggests a median age between 49 and 52 years, with some reports indicating a slight female predominance [32,34,35,37,40]. Although race is infrequently reported, ESPs appear more common among White patients [41]. A recent retrospective case–control study found that ESP patients were younger (median age 52 years, *p* = 0.021) but were more frequently African American (*p* < 0.001) and had a balanced male-to-female ratio compared to controls [3]. Similar findings were observed in an Italian cohort, where ESP patients were younger than the general EGD population, again without sex predominance [42]. 

Most ESPs are asymptomatic and incidentally discovered during EGD performed for unrelated reasons. When present, symptoms are typically non-specific and may include dyspepsia, heartburn, epigastric discomfort, or dysphagia. Due to their small size, ESPs rarely cause symptoms directly; instead, clinical manifestations are more often related to the underlying esophageal condition, such as reflux or inflammation. ESPs are most commonly located in the middle and distal thirds of the esophagus but may occur throughout the esophageal tract [3,32,37,43,44]. 

**Figure 1:**
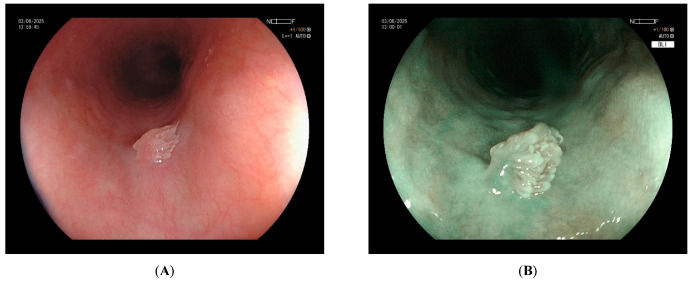
Endoscopic appearance of an esophageal squamous papilloma (ESP). (**A**): White light endoscopy (WLE) showing a small, solitary, whitish-pink lesion with well-demarcated margins. (**B**): Virtual chromoendoscopy using blue light imaging (BLI), highlighting the characteristic triad of exophytic growth, wart-like surface projections, and crossing vessels, features associated with high diagnostic accuracy.

**Figure 2:**
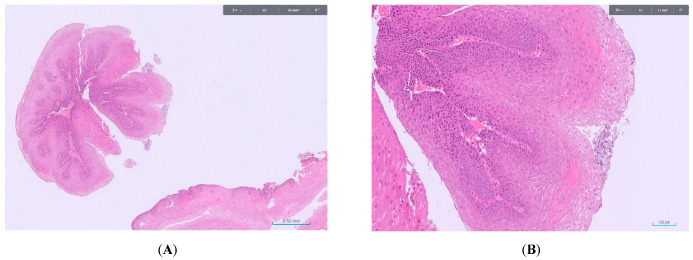
Histopathological features of esophageal squamous papilloma. (**A**): Low-power view (hematoxylin and eosin stain, 2×) showing exophytic finger-like projections arising from the esophageal mucosa. (**B**): Higher magnification (hematoxylin and eosin stain, 10×), highlighting the fibrovascular cores supporting the overlying stratified squamous epithelium.

Endoscopically, ESPs appear as a small (2–6 mm), solitary, whitish-pink, wart-like exophytic lesion—mostly pedunculated or semi-pedunculated—with well-demarcated margins. A definitive association between lesion morphology (pedunculated vs. sessile) and specific etiologic factors has not been established. Virtual chromoendoscopy techniques, including narrow-band imaging (NBI) and blue light imaging (BLI), improve diagnostic accuracy. One study reported that the triad of exophytic growth, wart-like projections, and crossing surface vessels had a positive predictive value of 88% [37] (Figure 1). Definitive diagnosis requires histologic confirmation. Histologically, ESPs consist of finger-like projections of squamous epithelium overlying a fibrovascular core (Figure 2). NBI often reveals brownish vascular lines corresponding to this fibrovascular core [37].

While most ESPs are solitary and small, rare cases of larger lesions (>10 mm), clustered lesions, or even diffuse esophageal involvement have been described. Extensive involvement is classified as esophageal squamous papillomatosis, a distinct entity associated with a higher likelihood of dysphagia and heartburn due to greater mucosal burden [45,46].

The exact pathogenesis of ESPs remains uncertain. Two major etiologic hypotheses have been proposed: chronic mucosal irritation and HPV infection [37,47]. 

Irritation-related factors include gastroesophageal reflux disease (GERD) [32,35,47], mechanical trauma [36] (e.g., sclerotherapy [48] and a self-expanding metal stent [49]), and alcohol and tobacco use [42]. GERD is supported by the frequent localization of ESPs in the distal esophagus [36]. Although reflux has been hypothesized as a contributing factor, no studies have reported reflux-associated histologic changes (e.g., basal cell hyperplasia or inflammation) in non-papillomatous mucosa of affected patients—an area requiring further investigation.

The association between HPV and ESPs was first suggested by Syrjanen et al. in 1982 [50]. Subsequent studies have reported HPV detection rates in ESPs ranging from 10 to 80% [32,35,39,47,51,52]. However, some authors failed to detect HPV in ESPs using either PCR or ISH [3,36,53,54,55]. 

Low-risk HPV types 6 and 11 are most commonly detected in HPV-positive ESPs [56]. For example, Takeshita et al. found HPV DNA (type 6) in 10.5% of samples [35]. High-risk HPV has also been identified. Odze et al [47] found HPV in 50% of ESPs examined, and subtype 16 was the most frequent. More recently, moreover, Tiftikçi et al. [52] showed that 19% of ESPs were positive for HPV DNA; three of them were of genotype 6, whereas four were of genotypes 16, 18, 31, and 81, which are known as highly oncogenic. Bohn et al. [32] reported that nearly 80% of ESPs were HPV-positive, mainly with low-risk types. In that study, HPV detection by amplified chromogenic ISH (ACISH) was comparable in sensitivity to PCR.

In conclusion, evidence increasingly supports an association between HPV infection and ESPs. However, many studies do not specify the HPV genotypes tested or limit analysis to common subtypes, likely underestimating true prevalence. Further research using broad-spectrum detection techniques is warranted to clarify this relationship and its clinical implications.

### 3.2. Esophageal Squamous Papillomatosis

While solitary esophageal squamous papillomas are rare, squamous papillomatosis of the esophagus is an even more exceptional condition. The literature is scarce and primarily consists of isolated case reports. The first described case was reported in 1977 in a 3.5-year-old boy with a pyriform sinus papilloma causing supraglottic obstruction [15]. 

Although sometimes grouped within the spectrum of EPSs, esophageal papillomatosis exhibits distinct clinical and pathological features. A recent review [2] of 53 published cases provided key epidemiological insights: the mean age of onset is approximately 46.8 years, with no significant sex-based or geographic differences. 

In contrast to solitary ESPs, which are typically asymptomatic, esophageal papillomatosis frequently presents with symptoms. Dysphagia was the most common complaint (32 out of 53 cases), followed by heartburn, weight loss, epigastric discomfort, and dyspepsia. In this context, the extent and confluence of lesions likely explain symptom onset, reflecting greater mucosal and inflammatory burden.

**Figure 3:**
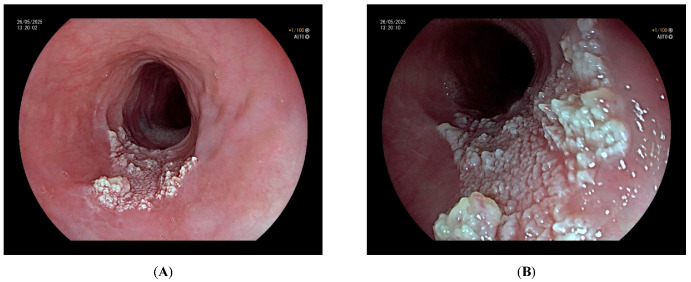
Endoscopic view of esophageal squamous papillomatosis under white light endoscopy (WLE). (**A**): Multiple closely packed, whitish mucosal projections with rough surfaces are visible, consistent with the characteristic appearance of extensive squamous papillomatosis. (**B**): A magnified view of the same lesion, allowing for a more detailed assessment of the papillary architecture and surface irregularities.

Diagnosis is made via EGD, where lesions typically appear as multiple small, whitish or pinkish projections with a smooth or slightly rough surface. They may be sessile or pedunculated and usually follow a tightly clustered pattern [57] (Figure 3). Lesion size can vary widely from 1 to 15 mm [58], and the number may be too high to count. Involvement may be limited to part of the esophagus, usually the middle and/or distal thirds, or extend along the entire esophageal tract, sometimes resulting in esophageal structures of varying degrees [2]. Advanced imaging techniques improve diagnostic accuracy. Narrow-band imaging with magnification [57,59,60], Lugol’s iodine staining [61,62], and endoscopic ultrasound (EUS) [63,64] are valuable tools. Despite suggestive endoscopic features, histologic confirmation remains essential. Typical histology reveals multiple finger-like projections of hyperplastic squamous epithelium overlying fibrovascular cores [57].

The pathogenesis of esophageal papillomatosis remains unclear, but it likely mirrors that of ESPs. Chronic mucosal irritation, especially due to GERD, is considered a major factor. Animal studies have shown that gastroesophageal and duodenal reflux can induce papillomatosis [65,66,67]. Other contributors include chemical irritants, such as tobacco and alcohol [35,62], and mechanical injuries, such as repeated esophageal dilations or metal stent placement [49].

These observations support the theory that chronic mucosal injury and subsequent regenerative hyperplasia play a key role in disease pathogenesis.

Evidence for HPV involvement in esophageal papillomatosis is limited. In the review by Li et al [2], HPV testing was performed in 29 of 53 cases, and 11 tested positive (37.9%). Among these, 12 HPV genotypes were identified; low-risk types 6 and/or 11 were present in six cases. Notably, four patients were positive for HPV 16, a high-risk genotype, half of whom developed esophageal cancer [68,69].

As in ESPs, the true prevalence of HPV in papillomatosis may be underestimated due to limited genotype screening and a lack of HPV testing in many reports.

## 4. Advanced Endoscopic Techniques for Lesion Characterization 

Endoscopy plays a central role in the detection, characterization, and surveillance of HPV-related esophageal squamous lesions. Given the documented risk of malignant transformation in esophageal squamous papillomas and papillomatosis, distinguishing benign from dysplastic or superficially invasive squamous cell carcinoma (ESCC) is critical for appropriate clinical management. Standard high-definition white light endoscopy (WLE) remains the first-line diagnostic tool, allowing for the evaluation of lesion location, morphology, size, and extent [2,37]. 

However, advanced imaging modalities, such as virtual chromoendoscopy, particularly narrow-band imaging (NBI) and blue light imaging (BLI), and dye-based chromoendoscopy, notably Lugol’s iodine staining, have demonstrated superior diagnostic performance in identifying early neoplastic changes, especially in superficial ESCC. These technologies enhance the ability to differentiate benign HPV-related lesions from those with dysplastic or malignant features [70]. 

### 4.1. Lugol’s Chromoendoscopy

Lugol’s iodine chromoendoscopy is a well-established technique for the early detection of squamous neoplasia. Iodine solution selectively stains glycogen-rich normal squamous epithelium dark brown, whereas dysplastic or neoplastic tissue—due to glycogen depletion—remains unstained or appears hypocolored, creating so-called "Lugol-voiding lesions." This contrast facilitates the identification of areas suspicious for dysplasia or carcinoma that may not be visible under WLE alone [71].

Lugol’s staining has proven useful in evaluating esophageal squamous papillomas and papillomatosis. Hypopigmented or unstained regions within lesions may indicate dysplasia or carcinoma. Several case reports have documented the successful application of Lugol's solution in delineating complex lesions and guiding targeted biopsies [57,61,62,70,72]. 

It is important to note that glycogen-depleted metaplastic epithelium (e.g., Barrett’s or heterotopic gastric mucosa) may also appear Lugol-negative, potentially mimicking dysplasia. Therefore, correlation with virtual chromoendoscopy and targeted biopsy is essential to avoid misinterpretation.

### 4.2. Virtual Chromoendoscopy (NBI and BLI) 

According to ESGE guidelines, virtual chromoendoscopy (NBI and BLI) is a validated alternative to Lugol’s chromoendoscopy for the detection of superficial ESCC. Both techniques outperform WLE alone in identifying early neoplasia [73,74]. 

Virtual chromoendoscopy enables detailed visualization of mucosal microvascular patterns, particularly intrapapillary capillary loops (IPCLs), which appear as brownish loops on magnified NBI and show characteristic morphological alterations in dysplastic or malignant tissue. Changes in IPCL shape, caliber, and distribution correlate with the degree of epithelial irregularity and neoplastic invasion. The IPCL classification system provides a morphological grading of these vascular changes. Types III to V—characterized by progressive dilation, tortuosity, and irregularity—are associated with high-grade dysplasia or carcinoma [75]. 

The JES (Japan Esophageal Society) classification further stratifies lesions based on IPCL features to estimate invasion depth as follows:

- Type A microvessels (without severe irregularity) suggest non-neoplastic or low-grade dysplastic lesions.
- Type B microvessels (with severe irregularity) indicate cancerous changes and are subdivided into the following:
  ○ B1: High-grade intraepithelial neoplasia or carcinoma limited to the mucosa (m1/m2).
  ○ B2: Invasion into muscularis mucosae (m3) or superficial submucosa (sm1).
  ○ B3: Invasion at least into deeper submucosa (≥sm2) [76,77,78].

The overall accuracy of type B vessels for predicting invasion depth exceeds 90% [79]. 

Although no studies have specifically evaluated the ability of virtual chromoendoscopy to differentiate esophageal squamous papillomas or papillomatosis from dysplasia or ESCC, several case reports suggest its utility. In benign papillomas, NBI typically reveals regular vascular patterns or subtle brownish vascular lines [37]. In contrast, dysplastic or malignant transformation is associated with irregular, dilated IPCLs. These observations support the value of NBI in improving diagnostic precision [57,59,60,72].

The integration of high-definition WLE, virtual chromoendoscopy (NBI/BLI), and Lugol’s staining offers a comprehensive endoscopic strategy. This multimodal approach improves lesion characterization, enhances risk stratification, and guides management decisions, particularly in HPV-related esophageal lesions that may harbor dysplastic or early neoplastic foci, especially in cases of extensive papillomatosis. 

Despite the enhanced sensitivity of these imaging techniques, histologic examination remains the diagnostic gold standard. Biopsy is essential, particularly in extensive or suspicious lesions. In esophageal papillomatosis, where focal dysplasia may be hidden within a macroscopically benign surface, multiple biopsies (typically two to fifteen) are recommended. Targeted sampling is especially important when suspicious features—such as irregular vascular patterns on NBI/BLI or hypopigmented areas on Lugol’s staining—are present, as these regions are more likely to harbor neoplasia [2].

## 5. Recurrence and Malignant Potential

Although esophageal squamous papilloma and papillomatosis are generally considered benign lesions, concerns have emerged regarding their recurrence and potential for malignant transformation, particularly in cases associated with hr-HPV infection. While conclusive evidence is lacking, an increasing number of studies have identified oncogenic HPV genotypes, especially HPV-16 and 18, not only in benign epithelial lesions but also in esophageal squamous cell carcinoma specimens. Recent meta-analyses have demonstrated a statistically significant association between HPV infection and ESCC, with odds ratios ranging from 2.7 to 3.8 [30,80,81,82]. These findings suggest a potential HPV-related continuum in esophageal pathology. A clearer understanding of recurrence patterns and malignant progression risk is essential to inform surveillance and therapeutic strategies.

### 5.1. Esophageal Squamous Papilloma

Esophageal squamous papilloma is generally regarded as a benign lesion and is typically managed with complete endoscopic resection. However, due to the rarity of this condition and the limited availability of long-term follow-up data, evidence on recurrence and malignant progression remains scarce [43]. 

Several studies have assessed clinical outcomes after ESP resection. In most cases, no recurrence was observed following the removal of benign, non-dysplastic lesions [3,34,36,83]. For example, a French study reported a 3.4% recurrence rate after the first ESP resection and 0% after the second [34]. Notably, some lesions were not fully excised during the initial procedure, raising the possibility that these recurrences may have represented residual disease rather than true recurrence. Similarly, in a recent retrospective case–control study, Ahmad et al. [3] found that among 18 patients who underwent follow-up endoscopy after ESP resection, 17 showed no evidence of recurrence.

However, follow-up adherence was suboptimal in all studies, with none reporting rates above 50%. This limitation hinders definitive conclusions about recurrence risk, although current evidence suggests that recurrence is rare or possibly absent in benign, non-dysplastic ESPs.

The malignant potential of ESPs remains controversial. While most lesions are considered non-neoplastic, isolated reports have described progression to ESCC. D’Huart et al [34] reported one case (1.3%) of malignant transformation among 78 patients, with SCC developing at the site of a previously resected low-grade dysplastic ESP two years earlier. The patient was a 75-year-old man with a history of smoking and alcoholic liver disease. Another case involved a 1.5 cm ESP at the esophagogastric junction that progressed to in situ carcinoma over two years in a 44-year-old woman without identifiable risk factors [84]. Similarly, in the study by Ahmad et al. [3], one of sixty-six patients developed ESCC during follow-up in the absence of known risk factors. Although these cases support a potential for malignant transformation, the absence of prospective studies and the limited follow-up periods preclude defining a specific timeframe between papilloma and carcinoma development. 

HPV has been implicated as a potential contributor to malignant progression based on its established role in cervical, anogenital, and oropharyngeal carcinogenesis [32]. However, HPV was not detected in the few ESP-associated ESCC cases reported, likely due to testing limitations or restriction to common genotypes (e.g., 6, 11, 16, 18) [3,34,84].

Although the oncogenic role of HPV in esophageal carcinogenesis remains controversial, the detection of high-risk genotypes in ESPs supports the hypothesis of a potential progression pathway [39,52]. This is further reinforced by growing evidence linking HPV to ESCC in certain populations.

### 5.2. Esophageal Squamous Papillomatosis

Esophageal squamous papillomatosis, although considered a benign condition, appears to carry a higher risk of malignant transformation than solitary ESPs. Macroscopic features such as large size, multiplicity, confluence, and circumferential distribution have been proposed as indicators of malignancy risk [34]. When extensive, this rare disease may pose both oncologic and quality of life concerns.

In a recent review, Li et al. [2] analyzed 53 reported cases of esophageal squamous papillomatosis and found that 12 patients (22.6%) developed ESCC [61,63,64,68,69,85,86,87,88,89,90,91]. In addition, three cases described varying degrees of dysplasia within the lesions [62,92,93]. Two more cases of low-grade dysplasia were reported in patients undergoing endoscopy for dysphagia [70,72]. These findings support the notion that esophageal papillomatosis may be a premalignant condition. Malignant transformation seems to occur within months to a few years after diagnosis, especially in cases with extensive mucosal involvement and high-risk HPV infection. However, precise estimates are limited by the scarcity of long-term follow-up and reliance on single case reports.

As in ESPs, HPV infection has been implicated in the malignant transformation of papillomatosis. Among the twelve cases that progressed to ESCC, only two had confirmed HPV infection [68,69]. Of the five dysplastic cases, only one tested positive for HPV [93]. In all three HPV-positive cases, the genotype identified was HPV-16. While these data suggest a possible oncogenic role, the association is not consistent. Additional cofactors, such as chronic gastroesophageal reflux, may be involved. Moreover, inconsistent HPV testing and limited genotype panels in many reports hinder interpretation. Despite this, the detection of high-risk HPV in cases progressing to dysplasia or carcinoma suggests a possible contributory role in oncogenesis.

Regarding recurrence, available data are limited. In the review by Li et al. [2], only nine of thirty patients (30%) treated with medical, endoscopic, or surgical approaches remained recurrence-free during follow-up (range: 3 months to 2 years). One illustrative case involved a patient with diffuse HPV 16-positive papillomatosis and HCV co-infection who underwent Ivor Lewis esophagectomy but experienced recurrence at the gastroesophageal anastomosis one year later [93]. This case underscores the importance of long-term surveillance and individualized therapeutic planning, even after radical treatment. 

In conclusion, while rare, esophageal squamous papillomatosis carries a measurable risk of malignant transformation and recurrence. The involvement of high-risk HPV genotypes suggests a possible progression pathway from benign epithelial proliferation to malignancy, similar to other HPV-associated epithelial conditions. Close clinical follow-up, personalized management strategies, and regular endoscopic monitoring are essential to detect progression at an early, treatable stage.

## 6. Treatment and Surveillance

Given the potential for dysplasia and malignant progression, esophageal squamous papillomas and papillomatosis should be completely removed when feasible [2,3]. Although specific clinical guidelines are lacking due to the rarity of these lesions, available data support a proactive approach to both treatment and surveillance.

### 6.1. Esophageal Squamous Papilloma

Despite their benign nature, esophageal squamous papillomas may carry a low risk of malignant transformation. Therefore, complete endoscopic removal is recommended. Most available data derive from case reports and small case series, and management should be tailored based on lesion size, morphology, histology, and patient risk factors.

Small (≤5 mm), pedunculated, or sessile lesions without dysplasia are typically managed with biopsy forceps [94,95,96,97]. Larger lesions (>5 mm) may require snare polypectomy or endoscopic mucosal resection (EMR) [40,84]. Endoscopic submucosal dissection (ESD) is reserved for lesions with suspicion of superficial ESCC (JES classification types A, B1, or B2), especially when en bloc resection is needed for accurate histologic staging [78,84].

Radiofrequency ablation (RFA) has also been reported as a potential treatment in selected cases [98]. Although not currently standard practice, it may offer a minimally invasive alternative, especially for small lesions, though it lacks the advantage of providing histologic samples.

Histopathological evaluation is essential for confirming the diagnosis and excluding dysplasia or carcinoma. Molecular testing for HPV may be considered, particularly in lesions with atypical features or dysplasia.

Surveillance should be individualized. Patients with larger lesions, dysplasia, superficial carcinoma, or high-risk HPV positivity may benefit from close endoscopic monitoring. In contrast, small, non-dysplastic, HPV-negative lesions completely removed may not require routine surveillance.

### 6.2. Esophageal Squamous Papillomatosis

Esophageal squamous papillomatosis carries a higher malignant potential than solitary papillomas, especially in extensive or multifocal disease. Treatment aims to eradicate lesions, relieve symptoms, such as dysphagia, and prevent malignant transformation. 

Various endoscopic modalities have been used, including argon plasma coagulation (APC), radiofrequency ablation (RFA) [99,100], cryotherapy [101,102], and photodynamic therapy (PDT) [86]. All four techniques have demonstrated efficacy in lesion treatment and potential prevention of malignant transformation. EMR [45] or ESD [70] may be employed for localized or suspicious lesions. ESD offers the advantage of en bloc resection and improved histologic assessment, particularly in the presence of dysplasia or high-risk features [78]. 

In select cases with malignant transformation or failure of endoscopic therapy, esophagectomy may be indicated [63,68,69,85,87,88].

Histologic and virologic assessment post-resection is crucial. 

Surveillance should be risk-adapted, taking into account lesion extent, HPV status, histological findings, and patient-specific factors. Chromoendoscopy during follow-up can aid in the detection of recurrent or progressing lesions. In the absence of dysplasia or high-risk HPV, conservative management with periodic monitoring may be reasonable, but no standardized surveillance protocols currently exist. 

Multidisciplinary evaluation and individualized follow-up remain key to optimal long-term outcomes. In particular, otolaryngologic assessment should be considered to rule out synchronous laryngeal or tracheal involvement, especially in pediatric patients or in cases of extensive or recurrent disease.

## 7. Conclusions

Esophageal squamous papilloma and esophageal squamous papillomatosis are rare epithelial lesions whose etiology, natural history, and optimal clinical management remain poorly defined. Current evidence suggests a possible etiological role of HPV infection, with low-risk genotypes more frequently associated with benign lesions and high-risk genotypes potentially involved in cases with dysplastic or malignant transformation. However, heterogeneity in study design, limited HPV genotyping, and non-standardized detection methods prevent definitive conclusions on the true oncogenic role of HPV in these lesions. 

Although malignant transformation appears infrequent, multifocality, lesion size, dysplasia, and high-risk HPV positivity may warrant closer surveillance. Complete endoscopic resection remains the treatment of choice, with histopathological and virological assessment guiding further management. 

Prospective, standardized studies are needed to clarify the natural history and optimize diagnostic and therapeutic algorithms.

## Abbreviations

The following abbreviations are used in this manuscript.

HPV	Human Papillomavirus
Hr-HPV	High-risk Human Papillomavirus
ESP	Esophageal Squamous Papilloma
RRP	Recurrent Respiratory Papillomatosis
ESCC	Esophageal Squamous Cell Carcinoma
PCR	Polymerase Chain Reaction
ISH	In Situ Hybridization
IHC	Immunohistochemistry
NBI	Narrow-Band Imaging
BLI	Blue Light Imaging
WLE	White Light Endoscopy
EGD	Esophagogastroduodenoscopy
GERD	Gastroesophageal Reflux Disease
ESD	Endoscopic Submucosal Dissection
EMR	Endoscopic Mucosal Resection
RFA	Radiofrequency Ablation
APC	Argon Plasma Coagulation
PDT	Photodynamic Therapy
IPCL	Intrapapillary Capillary Loop
JES	Japan Esophageal Society
ESGE	European Society of Gastrointestinal Endoscopy
